# Dual Emission in a Ligand and Metal Co-Doped Lanthanide-Organic Framework: Color Tuning and Temperature Dependent Luminescence

**DOI:** 10.3390/molecules25030523

**Published:** 2020-01-25

**Authors:** Despoina Andriotou, Stavros A. Diamantis, Anna Zacharia, Grigorios Itskos, Nikos Panagiotou, Anastasios J. Tasiopoulos, Theodore Lazarides

**Affiliations:** 1 Department of Chemistry, Aristotle University of Thessaloniki, 54124 Thessaloniki, Greece; despoina.andriotou95@gmail.com (D.A.); sdiamant@chem.auth.gr (S.A.D.); 2 Department of Physics, University of Cyprus, 1687 Nicosia, Cyprus; zacharia.anna@ucy.ac.cy (A.Z.); itskos@ucy.ac.cy (G.I.); 3 Department of Chemistry, University of Cyprus, 1687 Nicosia, Cyprus; panagiotou.nikos@ucy.ac.cy (N.P.); atasio@ucy.ac.cy (A.J.T.)

**Keywords:** metal-organic frameworks, luminescence, lanthanides, color tuning, doping, temperature sensors

## Abstract

In this study, we report the luminescence color tuning in the lanthanide metal-organic framework (LnMOF) ([La(bpdc)Cl(DMF)] (**1**); bpdc^2−^ = [1,1′-biphenyl]-4,4′-dicarboxylate, DMF = *N*,*N*-dimethylformamide) by introducing dual emission properties in a La^3+^ MOF scaffold through doping with the blue fluorescent 2,2′-diamino-[1,1′-biphenyl]-4,4′-dicarboxylate (dabpdc^2−^) and the red emissive Eu^3+^. With a careful adjustment of the relative doping levels of the lanthanide ions and bridging ligands, the color of the luminescence was modulated, while at the same time the photophysical characteristics of the two chromophores were retained. In addition, the photophysical properties of the parent MOF (**1**) and its doped counterparts with various dabpdc^2−^/bpdc^2−^ and Eu^3+^/La^3+^ ratios and the photoinduced energy transfer pathways that are possible within these materials are discussed. Finally, the temperature dependence study on the emission profile of a doped analogue containing 10% dabpdc^2−^ and 2.5% Eu^3+^ (**7**) is presented, highlighting the potential of this family of materials to behave as temperature sensors.

## 1. Introduction

The unique luminescence properties of trivalent lanthanide ions (Ln^3+^), including sharp atomic-like emission spectra, which are largely independent of the metal’s coordination environment and long lifetimes, reaching the order of a few milliseconds in the cases of Eu^3+^ and Tb^3+^, make them well suited as luminophores for a diverse range of applications spanning the fields of biotechnology, telecommunications, sensors and lighting [1,2,3,4]. Despite their favorable properties, the Laporte forbidden nature of f-f transitions makes luminescence through direct excitation of Ln^3+^ ions extremely inefficient. However, this shortcoming can be tackled through the coordination of Ln^3+^ ions to strongly absorbing chromophores which act as antennae by sensitizing metal-based emission through photoinduced energy transfer [5]. Recently, a considerable amount of research effort has been directed towards the development of lanthanide ratiometric thermometers [6] which are based on the temperature-induced changes in the photophysical behavior of at least two emission centers, thereby providing a more reliable and accurate self-referenced signal with reduced dependence on the experimental conditions. The majority lanthanide luminescent thermometers reported in the literature are based on measuring the ratio between the emission intensities of Tb^3+^ and Eu^3+^ centers at different temperatures [7,8,9,10] while those that involve the emission of a bridging ligand [11] or an encapsulated organic dye [12] are relatively rare.

In this contribution, we report the preparation and study of a homologous series of ligand and metal co-doped lanthanide-organic frameworks where a parent framework [La(bpdc)Cl(DMF)] (**1**) [13] is doped with the strongly fluorescent diamino derivative of the bpdc^2−^ bridging ligand dabpdc^2−^ and with the luminescent lanthanide ion Eu^3+^. The lanthanide MOF (LnMOF) ([La(bpdc)Cl(DMF)] (1) was chosen as a doping platform because: (i) its highly reproducible synthesis and chemical robustness, as dry crystals of **1** can be left in air for several months without showing any sign of deterioration; (ii) the bpdc^2−^ bridging ligand has been demonstrated to be a good sensitizer for the luminescence of the Eu^3+^ ion [14,15,16]; (iii) the possibility to obtain strong Ln^3+^ -based emission due to the absence of water from the coordination sphere of the Ln^3+^ ion which would provide an efficient non-radiative deactivation pathway for f-f excited states through vibrational coupling with O–H oscillators [17]. Thus, following the above mentioned doping procedure, we prepared an isostructural series of materials with the formula [La_1−x_Eu_x_(bpdc)_1−y_(dabpdc)_y_Cl(DMF)] (x = 0–0.025; y = 0 or 0.1) which show emission from both chromophores. With careful adjustment of the Eu^3+^ doping percentage while keeping the dabpdc^2−^ doping level at 10%, luminescence color tuning from blue to red through purple was achieved. In addition, a temperature dependent luminescence study of material 7 (x = 0.025; y = 0.1) shows that good temperature sensing action can be obtained in the region from 80 to 180 K with the sensitivity parameter reaching the maximum value 2.51 %K^−1^ at 80 K.

## 2. Results and Discussion

### 2.1. Synthesis and Structural Studies

**Figure 1:**
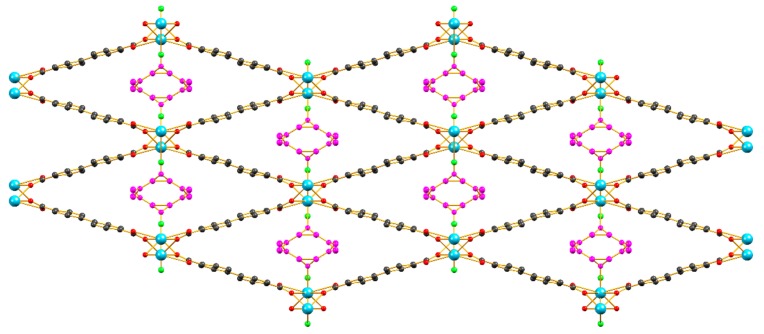

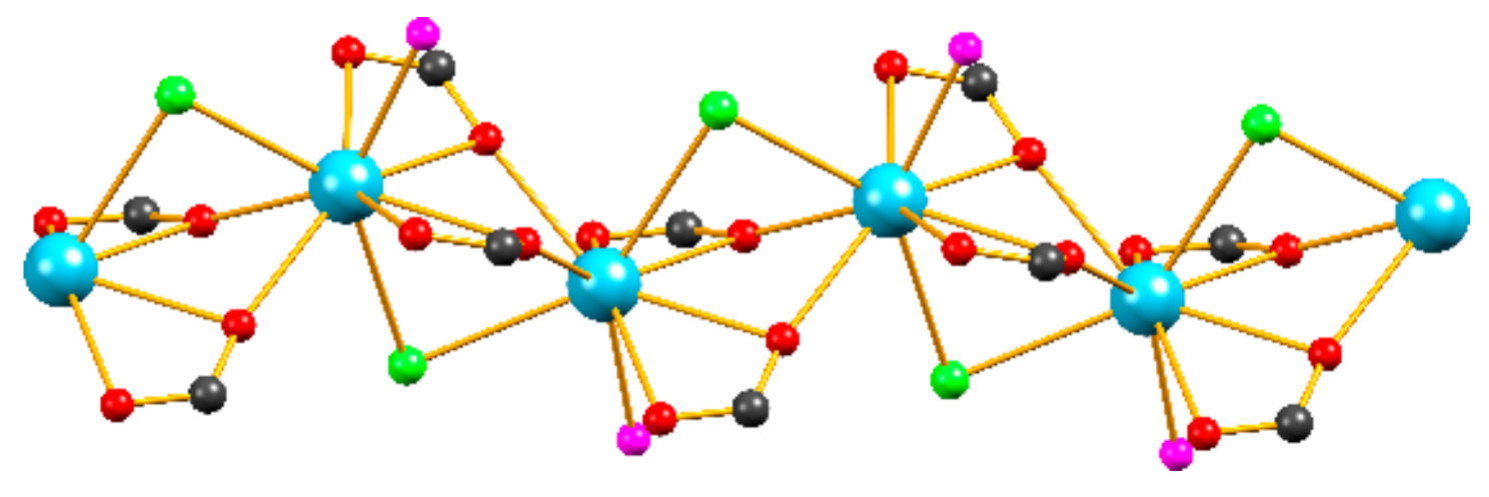
The crystal structure of the parent framework **1 [13]** viewed along the a axis. The infinite rod metal SBU of **1** is shown below the main structure. Color code: Lanthanum: turqoise, Carbon: black, Oxygen: red, Chlorine: green. The atoms of the coordinated DMF molecules are shown in magenta.

The reaction of the bridging ligand H_2_bpdc with LaCl_3_·xH_2_O in a 1:1 molar ratio in DMF at 110 °C, afforded a crystalline product **1** with the formula [La(bpdc)Cl(DMF)] which is isostructural to the compound of the same formula reported by Hou et al. in 2013 [13]. Compound **1** crystallizes in the orthorombic Pnma space group and features one crystallographically unique lanthanum cation with a coordination number of nine while its coordination polyhedron can be best described as a tricapped trigonal prism. As seen in Figure 1, the structure of **1** features an infinite rod secondary building unit (SBU) consisting of a zig-zag chain of La^3+^ ions bridged by μ_2_ Cl^−^ anions and by μ_2_-η^2^:η^1^ carboxylate units. Each bridging ligand is connected to two different chains, thus forming a three-dimensional framework with rhombic channels along the crystallographic axis which are occupied by terminally coordinated DMF molecules displaying two-fold positional disorder around the crystallographic mirror plane. Selected bond lengths and bond angles for **1** are listed in Supplementary Table S1.

**Figure 2:**
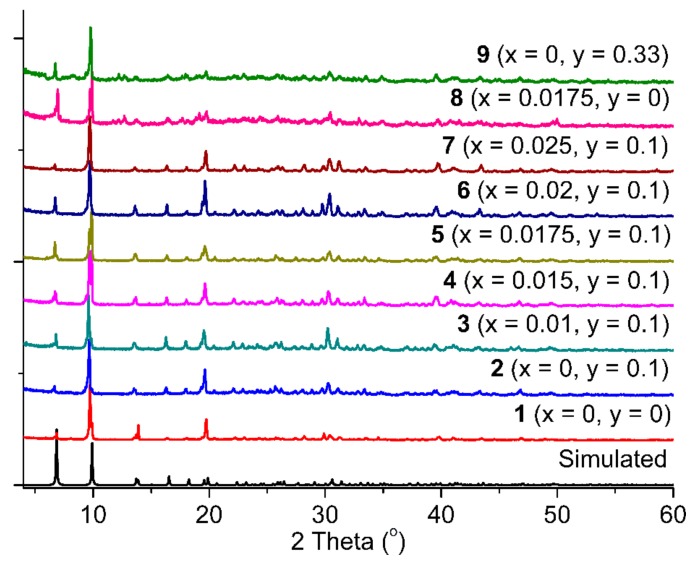
Powder X-ray diffraction paterns of the doped analogues **1**–**9** with the general formula [La_1−x_Eu_x_(bpdc)_1−y_(dabpdc)_y_Cl(DMF)]; the values of x and y corresponding to each doped analogue are shown on the graph.

In agreement with the findings of Hou et al. [13], frameworks isostructural to **1** could only be obtained with early lanthanide ions such as La^3+^, Pr^3+^ and Nd^3+^ while our attempts to prepare a luminescent Eu^3+^ analog of **1** were met with failure. We therefore decided to follow the strategy of metal doping in order to introduce the luminescent Eu^3+^ ion within the framework of **1**. Thus, a series of reactions in the presence 1–2.5 mol% of Eu^3+^ afforded crystalline products **2**–**9** which are isostructural to **1**, as confirmed by powder X-ray diffraction (pxrd) studies (Figure 2). The Eu^3+^ doped materials displayed the characteristic red luminescence of the Eu^3+^ ion upon being illuminated with a standard laboratory UV lamp (vide infra) thus showing that Eu^3+^ is successfully incorporated within the parent structure. This observation encouraged us to attempt further doping of the parent framework with the intensely blue fluorescent diamino derivative the H_2_bpdc bridging ligand 2,2′-diamino-[1,1′-biphenyl]-4,4′-dicarboxylic acid (H_2_dabpdc). Indeed, we found that the presence of up to 33 mol% of H_2_dabpdc in the initial reaction mixture leads to crystalline products which are isostructural to **1** (Figure 2). In order to gain better insight on the degree of incorporation of dabpdc^2−^ within the parent framework of **1**, we subjected a sample of **2** (0 mol% Eu^3+^ and 10 mol% of H_2_dabpdc in the reaction feed) to ^1^H-NMR analysis after it was digested in a mixture of D_2_O/NaOH. From the ratio of the peak integrals corresponding to bpdc^2−^ and dabpdc^2−^ (Supplementary Figure S1), we calculated a molar fraction of 12% of dabpdc^2−^ within **2** which is close to the molar percentage of dabpdc^2−^ present in the reaction feed. This finding suggests that, in the employed experimental conditions, dabpdc^2−^ is similar to bpdc^2−^ in terms of reactivity towards La^3+^ and at least relatively low percentages of dabpdc^2−^ in the reaction feed result in a statistical distribution of the amino substituted bridging ligand within the product.

**Figure 3:**
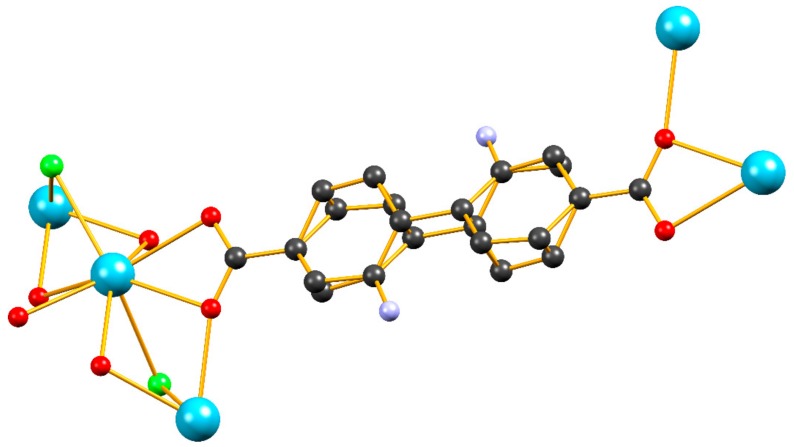
Partial view of the crystal structure of **9** highlighting the disorder of the biphenyl bridging unit. The nitrogen atoms were refined with a site occupancy of ca. 17%. Hydrogen atoms and the coordinated DMF molecules are omitted for clarity. Color code: Lanthanum: turqoise, Carbon: black, Oxygen: red, Nitrogen: blue, Chlorine: green.

In addition to the ^1^H-NMR study, we carried out single crystal X-ray structural analysis on a sample of **9** (0 mol% Eu^3+^ and 33 mol% of H_2_dabpdc in the reaction feed). Crystal and refinement data can be found in Supplementary Table S2. The overall structure of **9** is virtually identical to that of **1** with the difference that the bridging ligand shows significantly greater disorder and had to be refined in two positions (Figure 3). We were also able to locate one of the two nitrogen atoms of dabpdc^2−^ which refined well with a given site occupancy of ca. 17%. In addition, several constraints were applied in order to keep the C–N distance and the angles around the C–N bond within chemically acceptable values. The disorder of the bridging ligand in **9** is possibly a result of the different conformations adopted by bpdc^2−^ and dabpdc^2−^_._ In the parent compound **1**, the bpdc^2−^ ligand adopts a conformation where the two phenylene groups of the biphenyl spacer are virtually co-planar [13] a feature that is commonly found in structures containing the bpdc^2−^ ligand [18,19,20,21,22,23,24,25,26] while, as observed by us [27] and others [28], the dabpdc^2−^ bridging ligand tends to adopt a staggered syn conformation where the dihedral angle between the two phenylene groups is in the order of 60–70^o^. It is therefore reasonable to expect that the presence of about one third dabpdc^2−^ at the sites normally occupied by bpdc^2−^ in the parent framework would induce some additional disorder to the diphenylene spacer. Consequently the ^1^H-NMR and crystallographic data indicate that even though the dabpdc^2−^ moiety seems to have slightly different stereochemical demands than the bpdc^2−^ ligand of the parent framework, its incorporation does not induce a big distortion to the overall structure.

### 2.2. Thermogravimetric Analysis

**Figure 4:**
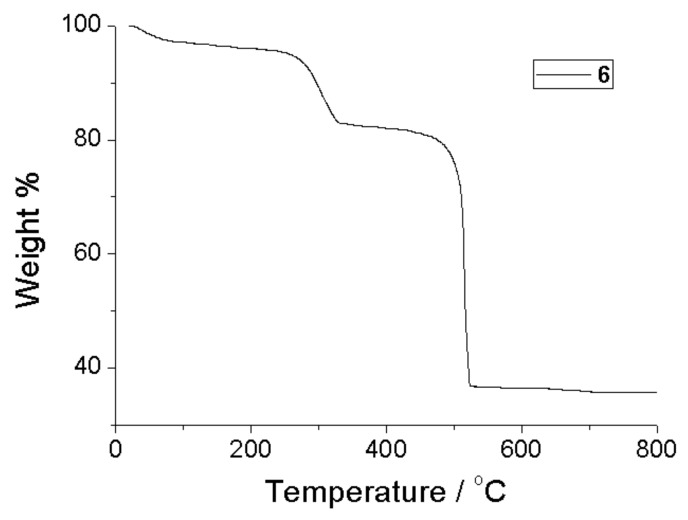
The TGA curve of compound **6**.

The thermal stability of **1**, **2** and **6** was studied by thermogravimetric analysis (TGA) under air (see Supplementary Figures S2 and S3). All the analogues show essentially identical behavior and for this reason only the thermograph of **6** (Figure 4) shall be discussed. In particular, the weight loss in **6** was observed in two steps. The first step is observed in the temperature range 240–330 °C and the corresponding weight loss is attributed to the coordinated DMF molecules (experimental loss: 16.83%, theoretically estimated loss of 14.9%). The framework remains thermally stable up to ~500 °C and then the second mass loss step appears, corresponding to the decomposition of the framework, which is completed up to ~525 °C.

### 2.3. Luminescence Properties

**Figure 5:**
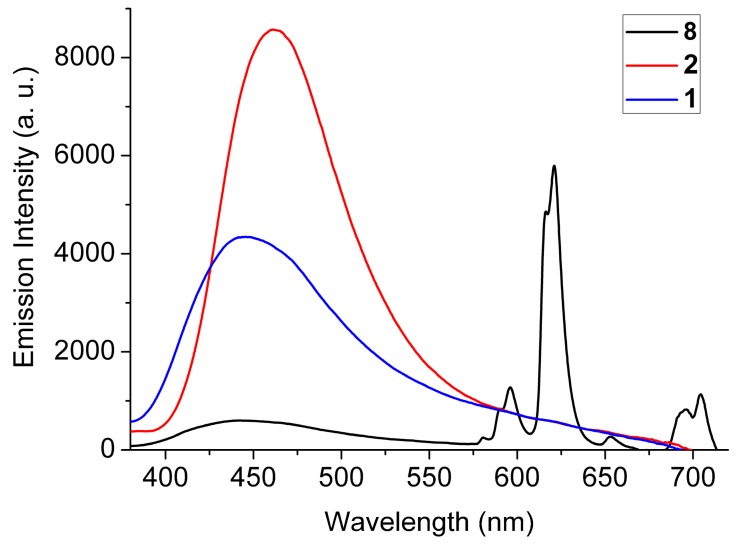
The solid-state emission spectra of compounds **1**, **2** and **8** upon excitation at 365 nm. See main text for details.

Photophysical studies on microcrystalline powders of the parent framework **1** and its metal and ligand doped counterparts were carried out by emission spectroscopy. Excitation of compound 1 at λ_exc_ = 365 nm gives rise to a fluorescence band with maximum at ca. 440 nm (Figure 5), which is attributed to the radiative deactivation of the lowest energy ^1^π-π* excited state of the bpdc^2−^ bridging ligand [13,14]. The excitation spectrum of **1** (monitored at 450 nm) shows that the lowest energy absorption feature is at 375 nm and tails off rapidly after 400 nm, thereby showing virtually no absorption in the visible region (Figure 5). When the parent framework of **1** is doped 10 mol% with the diamino derivative dabpdc^2−^ (2), a rather small red shift in the fluorescence peak which maximizes at ca. 463 nm was observed. Based on a comparison with our previous work on the fluorescence properties of Ca^2+^ and Sr^2+^ MOFs featuring (NH_2_)_2_bpdc^2−^ as bridging ligand, we attribute the red shifted emission signal of **2** predominantly to the fluorescence from the dabpdc^2−^ chromophore [27]. From the onsets of the emission peaks of the two chromophores in **1** and **2** the energies of the lowest lying ^1^π-π* of excited states of bpdc^2−^ and dabpdc^2−^ were estimated at ca. 25,000 and 23,500 cm^−1^ respectively [29]. It therefore follows that initial excitation of predominantly the bpdc^2−^ moiety of **2** (mainly due to its much higher abundance within the material’s framework) is followed by energy transfer to the dabpdc^2−^ chromophore most possibly through a mechanism involving exciton diffusion to a position adjacent to a dabpdc^2−^ group and subsequent coulombic (Förster) energy transfer to the latter [30,31,32,33,34]. It is important to mention that the emission profiles of samples doped with significantly larger percentages of dabpdc^2−^ (such as sample **9**) are virtually identical to that of **2**, thus confirming that in the latter material interchromophore energy transfer reaches its maximum efficiency.

The emission spectrum of compound 8 (λ_exc_ = 365 nm), where the parent framework of **1** is doped only with 1.75 mol% Eu^3+^, is dominated by the Eu^3+^-based ^5^D_0_ → ^7^F*_J_* (*J* = 0–4) emission peaks which are located at 580, 596, 620, 654 and 704 nm respectively. On the high energy region of the spectrum, we observe the relatively weak residual emission of the bpdc^2−^ bridging ligand which maximizes at ca. 440 nm indicating that, even at this relatively low doping level of 1.75 mol%, ligand-to-Eu^3+^ energy transfer is quite efficient [1,35]. The fact that the bpdc^2−^ bridging ligand is a good sensitizer for both dabpdc^2−^ and Eu^3+^ prompted us to synthesize and study frameworks where both energy acceptors are present within the parent framework of **1**.

**Figure 6:**
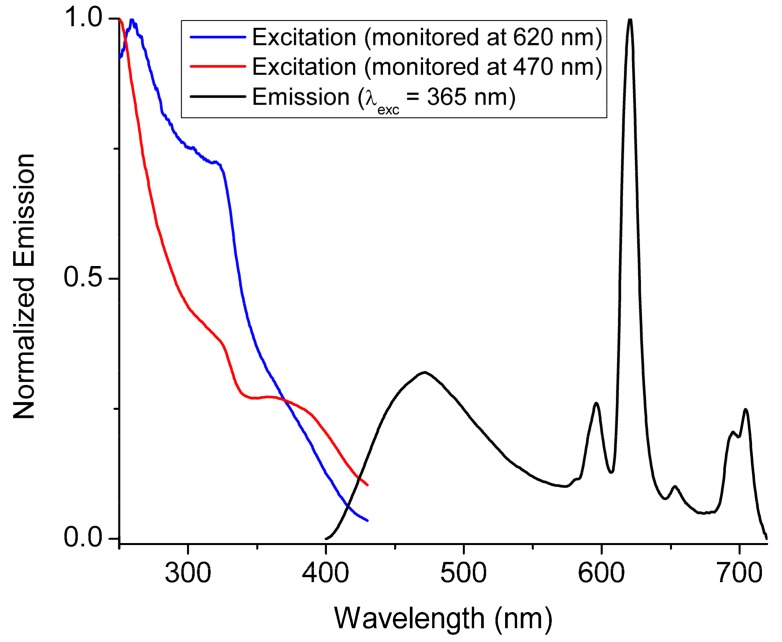
The solid-state emission and excitation spectra of compound 6. The excitation spectra are monitored both at the dabpdc^2−^ (470 nm) fluorescence and the Eu^3+^ emission (620 nm).

In the case of **6**, where both dabpdc^2−^ and Eu^3+^ are doped into the parent framework of **1**, excitation at 365 nm results in emission from both the amino substituted organic chromophore and the luminescent lanthanide ion (Figure 6). The maxima of the ^5^D_0_ → ^7^F*_J_* (*J* = 0−4) peaks of Eu^3+^ in **6** are in the expected positions while the fluorescence from the dabpdc^2−^ chromophore occupies the blue region of the spectrum showing a maximum at ca. 470 nm (vide supra). The excitation spectra of **6** were measured monitoring at both the ligand (470 nm) and Eu^3+^ (620 nm) emissions and are shown in Figure 6. We observe that upon monitoring the dabpdc^2−^ ligand emission, the excitation spectrum of **6** is dominated by the absorption features of the bpdc^2−^ chromophore, while monitoring at the Eu^3+^ emission results in an excitation spectrum showing a relatively weak albeit clear shoulder at ca. 425 nm which tails off above 445 nm. This spectral feature is attributed to the absorption of the dabpdc^2−^ chromophore [27] and indicates that the latter may also sensitize Eu^3+^ emission.

**Figure 7:**
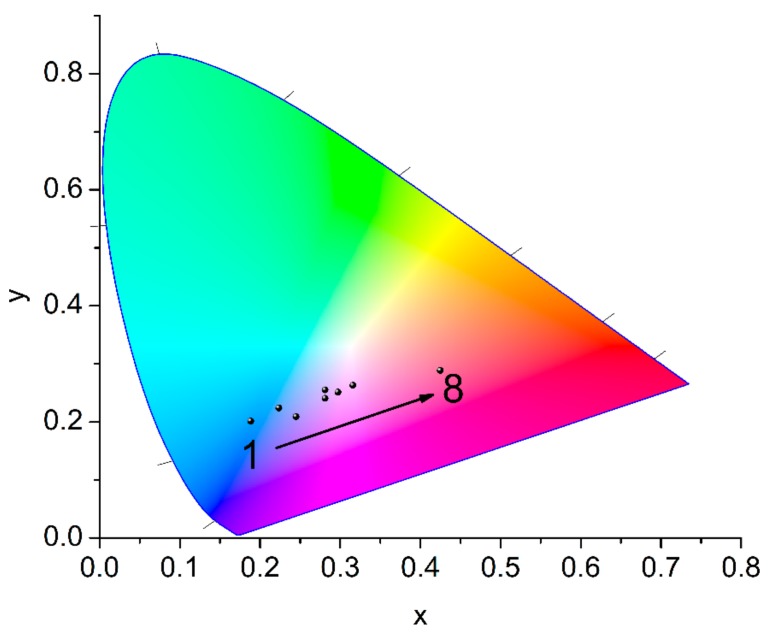
Chromaticity coordinates (CIE 1931) calculated from the corrected emission profiles of materials **1**–**8** showing the gradual shift from blue to red upon increasing the Eu^3+^ content.

Starting from **2** and progressively doping the framework with increased levels of Eu^3+^ (*vide supra*) results in increased lanthanide-based emission with a concomitant decrease of the contribution from the organic chromophore. The change in color of the materials doped with 10 mol% of dabpdc^2−^ and increasing levels of Eu^3+^ (0–2.5 mol%) is demonstrated by the CIE (Commission Internationale de l’Éclairage) coordinates of the chromaticity diagram of Figure 7, where we see that the emission color gradually shifts from the blue (x = 0.172, y = 0.173 for **2**) to the purple-red region of the spectrum (x = 0.425, y = 0.289 for **8**). However, the absence of a strong yellow-green component in the emission spectra of this series of materials does not allow sufficient color tuning in order to achieve entry in the white region (x and y values of 0.3 and above) [36]. Instead, a further increase of the Eu^3+^ doping levels results in the emission color traversing the purple region and eventually entering the red region.

**Figure 8:**
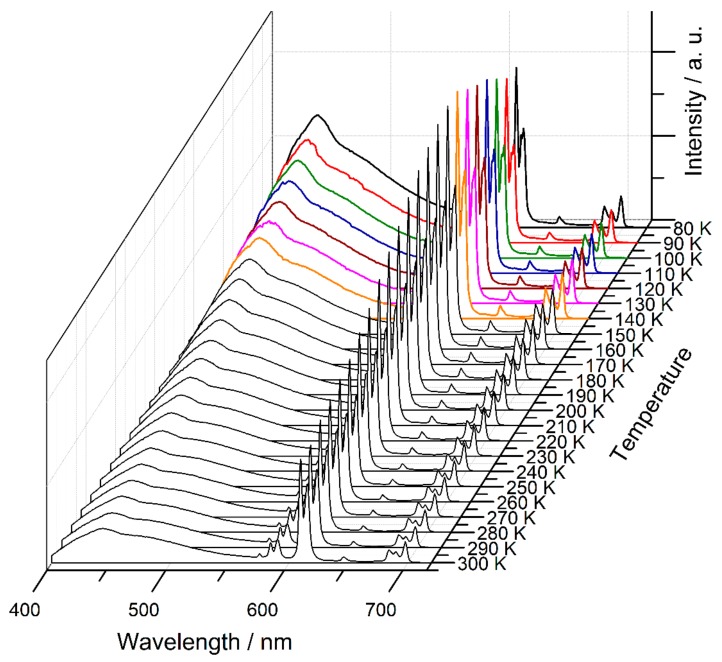
Temperature dependent emission spectra of **7** upon excitation at 375 nm. The spectra in the temperature region between 80 and 140 K are highlighted to emphasize the increase in Eu^3+^-based emission with rising temperature (see main text).

Nonetheless, the presence of two clear emission components in **7**, arising from an organic chromophore and a lanthanide, prompted us to study the effect of temperature on its emission profile in order to explore the potential of the material to perform as a ratiometric luminescence thermometer [8,37,38,39,40]. Figure 8 shows the emission spectra of **7** at various temperatures from 80 to 300 K. By examining the spectra of Figure 8, we observe that the ligand component shows a steady decrease in intensity with rising temperature while the Eu^3+^ luminophore shows distinctly different behavior in two different temperature regions. In the region between 80 and ca. 140 K, we observe an increase of Eu^3+^ emission intensity, while in the region between 150 and 300 K, the emission intensity of Eu^3+^ follows the steady decrease of that of the organic component. The decrease in the fluorescence intensity of the organic component as the temperature rises can be mainly attributed to the increasing participation of non-radiative pathways in the decay process of the dabpdc^2−^ chromophore [29]. The initial rise of the Eu^3+^ component between 80 and 150 K possibly indicates that, at that temperature range, ligand-to-metal energy transfer is a major non-radiative deactivation pathway for the dabpdc^2−^ excited state. At higher temperatures, increased molecular and lattice vibrations render thermal deactivation pathways dominant, and thereby lead to the observed reduction in the intensities of both the organic and Eu^3+^ emission components.

**Figure 9:**
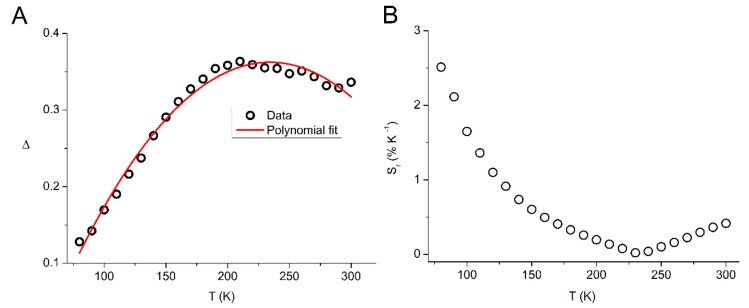
(**A**) The thermometric parameter versus temperature for material **7**. The red line represents a polynomial fit to the experimental data. (**B**) The $S_{r}$ parameter versus temperature for material **7**. See main text for details.

If we define the ratio of the integrated intensities of the Eu^3+^-based ^5^D_0_ → ^7^F*_2_* emission peak ($I_{Eu}$) to the ligand-based fluorescence signal ($I_{L}$) as the thermometric parameter Δ and plot the result against the temperature T, we obtain the diagram of Figure 9. The data can be fitted satisfactorily (correlation coefficient R^2^ = 0.984) to a second-degree polynomial (Equation (1)).

(1) $$\Delta \;=\;-1.05\;\times \;10^{-5}\;T^{2}\;+\;4.90\times 10^{-3}\;T\;-\;0.21$$

The performance of a temperature sensor is often reported in terms of its relative sensitivity [41], $S_{r}$, which serves as a figure of merit to allow comparison between different temperature sensors reported in the literature and is defined in Equation (2) [8,42].

(2) $$S_{r}=\frac{1}{\Delta }\left|\frac{\partial \Delta }{\partial Τ}\right|$$

The $S_{r}$ parameter of **7** as %K^−1^ is plotted against the temperature in Figure 9B. The maximum value of the relative sensitivity $S_{m}$ = 2.51 %K^−1^ is obtained at 80 K. These results indicate that **7** can function as a ratiometric luminescence thermometer in the tested region from 80 to 300 K showing its best performance in the 80 to 150 K region. The maximum relative sensitivity of 2.51 %K^−1^ shown by **7** compares well with the typical values obtained with many luminescent thermometers based on lanthanide organic frameworks [8,9,10,43]. Therefore, combined ligand and metal doping can be an effective route for the preparation of improved luminescent temperature sensors.

## 3. Conclusions

We demonstrated that the parent framework of [La(bpdc)Cl(DMF)] (**1**) [13] can be easily doped with the bridging ligand, dabpdc^2−^ (the diamino derivative of bpdc^2−^ ligand present in **1**), and Eu^3+^ to produce a range of mixed ligand and mixed metal analogues. Doping of ca. 10 mol% with dabpdc^2−^ (**2**) results in a moderate red shift of the ligand-based fluorescence due to interligand photoinduced energy transfer. Further doping with various amounts of Eu^3+^ yields materials which display sensitized Eu^3+^ emission along with the blue dabpdc^2−^ fluorescence. Emission color tuning was achieved by varying the Eu^3+^ doping percentage from 0 to 2.5 mol%, from blue to red through purple. However, the absence of a yellow-green component in the emission spectra of this series did not allow the achievement of white light luminescence. Finally, we studied the temperature dependence of the emission profile of **7** (10 mol% dabdc^2−^, 2.5 mol% Eu^3+^) in the region from 80 to 300 K. While the ligand component shows a steady decrease in fluorescence intensity with increasing temperature, the Eu^3+^ luminescence shows an initial enhancement from 80 to ca. 150 K before following the trend of the organic portion of the emission spectrum of **7**. We attribute the initial enhancement of Eu^3+^ luminescence to the dominance of ligand-to-metal energy transfer over thermal decay pathways at relatively low temperatures. Compound **7** shows good potential as a ratiometric luminescence thermometer displaying its best performance in the 80 to 150 K region with maximum sensitivity of 2.51 %K^−1^ at 80 K. This result shows that combined ligand and metal doping can be a viable route to produce new luminescence-based temperature sensors. Our group is currently working towards the construction of mixed lanthanide–organic frameworks exhibiting white luminescence and luminescence-based temperature sensing properties by following the above described strategy. The results obtained from the current study can be a stepping-stone towards the construction of superior optical materials.

## 4. Materials and Methods

### 4.1. Synthesis

Starting materials and solvents were purchased from the usual commercial sources (Sigma-Aldrich and Alfa Aesar) and were used as received.

#### 4.1.1. Synthesis of H_2_dabpdc

*Dimethyl-2,2′-dinitro-[1,1′-biphenyl]-4,4′-dicarboxylate.* Dimethyl-biphenyl-4,4′-dicarboxylate (1.00 g, 3.7 mmol) was added into concentrated H_2_SO_4_ (10 mL). The mixture was stirred at room temperature for 10 min. Nitric acid (760 μL, 3 eq.) was added into concentrated H_2_SO_4_ (2 mL). This solution was added dropwise into the first mixture at room temperature over a period of 20 min. The mixture was stirred at room temperature for 4 h and then poured into ice (300 mL) to form a beige solid. The resulting solid was dissolved in dichloromethane and the aqueous phase was extracted with dichloromethane (3 × 70 mL). The combined organic layers were dried over Na_2_SO_4_ and evaporated under reduced pressure to afford a beige solid. The crude mixture was recrystallized from 2-propanol and washed with diethyl ether to give the pure product. Yield: 1.2 g (3.33 mmol, 90%). ^1^H NMR (500 MHz, CDCl_3_): δ (ppm): 8.90 (s, 2H), 8.37 (d, *J* = 8.4 Hz, 2H), 7.40 (d, *J* = 8 Hz, 2H), 4.02 (s, 6H).

*Dimethyl-2,2′-diamino-[1,1′-biphenyl]-4,4′-dicarboxylate.* Dimethyl-2,2′-dinitro-[1,1′-biphenyl]-4,4′-dicarboxylate (1.2 g, 3.33 mmol) was dissolved in 20 mL acetic acid and the solution was stirred under Ar atmosphere for 10 min. To this solution was added iron powder (3.7 g, 10 eq.) and the resulting mixture was stirred at room temperature for 24 h. The suspension was filtered through celite, washed with 40 mL acetic acid and the filtrate was concentrated under reduced pressure. The solid was dissolved in ethyl acetate (80 mL) and was extracted with saturated aqueous sodium carbonate solution (2 × 80 mL) and H_2_O (3 × 80 mL). The combined organic layers were dried over Na_2_SO_4_ and the solution was evaporated under vacuum to yield the product as a yellow solid. Yield 900 mg (3 mmol, 90%). ^1^H NMR (500 MHz, DMSO-*d*_6_): δ (ppm): 7.42 (s, 2H), 7.21 (d, *J* = 7.8 Hz, 2H), 7.06 (d, *J* = 7.8 Hz, 2H), 4.97 (s, 4H), 3.81 (s, 6H).

*2,2′-Diamino-[1,1′-biphenyl]-4,4′-dicarboxylic acid (H_2_dabpdc).* Dimethyl 2,2′-diamino-[1,1′-biphenyl]-4,4′-dicarboxylate (900 mg, 3.0 mmol) was dissolved in THF (20 mL) and 20 mL of aqueous NaOH (0.6 M) were added dropwise under vigorous stirring. The mixture was stirred overnight at room temperature. The organic solvent was removed under vacuum and the aqueous solution was acidified with acetic acid to yield a light brown solid (735 mg, 2.7 mmol, 90%). ^1^H-NMR (500 MHz, DMSO-*d*_6_): δ (ppm) = 12.64 (br, 2H), 7.40 (s, 2H), 7.20 (d, *J* = 7.6 Hz, 2H), 7.40 (d, *J* = 7.8 Hz, 2H), 4.90 (br, 4H).

#### 4.1.2. Synthesis of MOFs

Synthesis of [La(bpdc)Cl(DMF)] (**1**). LaCl_3_·7H_2_O (74.8 mg, 0.2 mmol) and biphenyl-4,4′-dicarboxylic acid (48.4 mg, 0.2 mmol) were added in DMF (3 mL) and stirred until the solids were fully dissolved. The resulting solution was sealed in a screw cap 23 mL scintillation vial and placed in a preheated oven at 110 °C where it remained undisturbed for 24 h before being cooled to room temperature. Colorless needle-like crystals were isolated by filtration, washed with DMF (5 × 3 mL), and dried under vacuum overnight. Yield 32 mg (45%).

Synthesis of the [La_1−x_Eu_x_(bpdc)_1−y_(dabpdc)_y_Cl(DMF)] series. LaCl_3_·7H_2_O (74.8 mg, 0.2 mmol) and biphenyl-4,4′-dicarboxylic acid (48.4 mg, 0.2 mmol) were added in DMF (3 mL) and stirred until the solids were fully dissolved. Standard solutions of EuCl_3_·6H_2_O (10^−2^ M) and H_2_dabdc (10^−2^ M) in DMF were prepared and calculated volumes were added in the reaction feed using a volumetric pipette, while the mixture was magnetically stirred, to achieve the desired La^3+^/Eu^3+^ and H_2_bpdc/H_2_dabpdc molar ratio. Otherwise, the procedure was identical to that for the synthesis of **1**. Colorless or pale-yellow needle-like crystals were isolated by filtration, washed with DMF (5 × 3 mL) and dried under vacuum overnight. Exact percentages of Eu^3+^ and dabpdc^2−^ in each doped analogue and reaction yields are shown in Table 1.

### 4.2. Physical Measurements and Crystallogtraphy

Photoluminescence spectra. The emission spectra were measured on a Horiba fluorescence spectrometer equipped with a powder sample holder. The light source was a 450 W Xenon Arc Lamp (220–1000 nm) and the detector a red sensitive Hamamatsu R928 photomultiplier tube. All spectra were corrected for instrument response using the correction function generated after calibration of the instrument with a standard light source. Appropriate long pass filters were used to remove scattering from the sample and the monochromators. Temperature-dependent photoluminescence measurements were carried out in the 80–300 K range by placing the samples in the cold finger of a Janis VPF liquid nitrogen optical cryostat.

^1^H-NMR. ^1^H-NMR spectra were recorded at room temperature on NMR Agilent 500 MHz, with the use of the solvent proton as an internal standard and on an Avance Brucker NMR spectrometer (500 MHz).

PXRD measurements. PXRD diffraction patterns were recorded on a Shimadzu 6000 Series X-ray diffractometer with a Cu K_α_ source (λ = 1.5418 Å).

X-ray Crystal Structure Determination. Single crystal X-ray diffraction data were collected on a Rigaku Oxford-Diffraction Supernova diffractometer, equipped with a CCD area detector utilizing Cu Kα (λ = 1.5418 Å) radiation. A suitable crystal was mounted on a Hampton cryoloop with Paratone-N oil and transferred to a goniostat where it was cooled for data collection. Empirical absorption corrections (multiscan based on symmetry-related measurements) were applied using CrysAlis RED software [44]. The structure was solved by direct methods using SIR2004 [45] and refined on F^2^ using full-matrix least-squares with SHELXL-2014/7 [46] within the WinGX [47] platform. Software packages used were as follows: CrysAlis CCD for data collection [44], CrysAlis RED for cell refinement and data reduction [44], and MERCURY [48] for molecular graphics. The non-H atoms were treated anisotropically, except for those that belong to disordered parts. The aromatic H atoms were placed in calculated, ideal positions and refined depending on their respective carbon atoms. Selected crystal data for **9** are summarized in Table S2.

Thermogravimetric Analysis (TGA). Thermal stability studies were performed with a Shimadzu TGA 50 thermogravimetric analyzer. Thermal analysis was conducted from 25 to 800 °C under air with a heating rate of 10 °C min^−1^.

## Figures and Tables

**Table 1:** Percentages of H_2_dabpdc and Eu^3+^ doping ^1^ and yields.

Compound	mol% H_2_dabpdc	mol% Eu^3+^	Yield
**2**	10	0	42
**3**	10	1.00	41
**4**	10	1.50	40
**5**	10	1.75	45
**6**	10	2.00	41
**7**	10	2.50	44
**8**	0	1.75	39
**9**	33	0	32

^1^ Molar percentage of dopant in the reaction mixture.

## Supplementary Materials

The following are available online, Table S1: Selected bond lengths and angles for 1., Table S2: Crystal and refinement data for 9, Figure S1: 1H-NMR spectrum of a digested sample of 2 in D2O/NaOH., Figure S2: The TGA curve for 1, Figure S3: The TGA curve for **2**.
