# Supplementary material for: Dual Emission in a Ligand and Metal Co-Doped Lanthanide-Organic Framework: Color Tuning and Temperature Dependent Luminescence
Source: Molecules. 2020 Jan 25;25(3):523. doi: 10.3390/molecules25030523 (PMC7037827; doi:10.3390/molecules25030523)
Supplement: Supplementary file 1 [file molecules-25-00523-s001.pdf]

Supplementary Information

Dual Emission in a Ligand and Metal Co-Doped Lanthanide-Organic Framework: Color Tuning and Temperature Dependent Luminescence

Despoina Andriotou [1], Stavros A. Diamantis [1], Anna Zacharia [2], Grigorios Itskos [2], Nikos Panagiotou [3], Anastasios J. Tasiopoulos [3] and Theodore Lazarides [1,*]

[1] Department of Chemistry, Aristotle University of Thessaloniki, 54124 Thessaloniki, Greece; despoina.andriotou95@gmail.com (D.A.); stvrosdiamantis@gmail.com (S.A.D.)

[2] Department of Physics, University of Cyprus, 1687 Nicosia, Cyprus; zacharia.anna@ucy.ac.cy (A.Z.); atasio@ucy.ac.cy (G.I.)

[3] Department of Chemistry, University of Cyprus, 1687 Nicosia, Cyprus; panagiotou.nikos@ucy.ac.cy (N.P.); atasio@ucy.ac.cy (A.J.T.)

[*] Correspondence: tlazarides@chem.auth.gr; Tel.: +30-2310-997853

Table S1. Selected bond lengths and angles for **1.**

Bond lengths	(Å)	Angles	(°)
La1-Cl1	2.988	La1-Cl1-La1′	86.30
La1′-Cl1	2.862	La1-O1-La1′	102.70
La1-O1	2.608	O1-La1-O2	50.05
La1-O2	2.570	Cl1-La1-Cl1′	141.65
La1-O3	2.463	O1-La1-O1′	68.07
La1′-O1	2.515	O1-La1-O2	50.05

Table S2. Crystal and refinement data for **9**.

Chemical formula	$C_{17}H_{6.37}ClLaN_{1.19}O_5$
Formula mass	481.58
Crystal description	block
Crystal size (mm)	0.071; 0.021; 0.018
Crystal system	orthorhombic
a(Å)	7.4701(4)
b(Å)	25.7440(14)
c(Å)	9.4625(7)
α(°)	90
β(°)	90
γ(°)	90
Unit cell Vol (Å^3)	1819.74(19)
Temperature (K)	100
Space group	P n m a
Z	2
No. of reflections measured	7804
No. of unique reflections	2240
No. of parameters/restraints	147/43
Restrained GooF	1.104
R_{int}	0.050
$R[F^2 > 2\sigma(F^2)]$	0.045
R[All data]	0.062
$wR(F^2)$	0.125

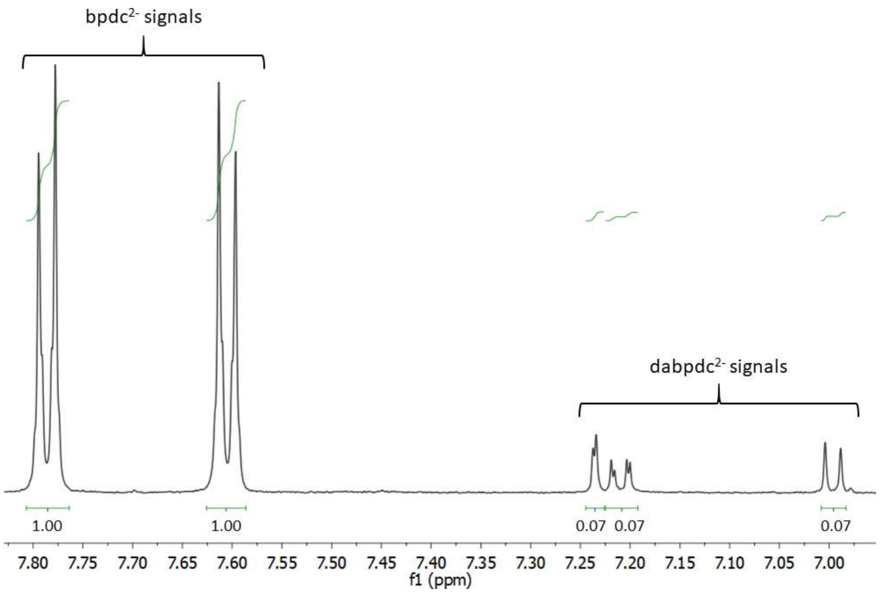

Figure S1. ¹H-NMR spectrum of a digested sample of **2** in D_2O/NaOH.

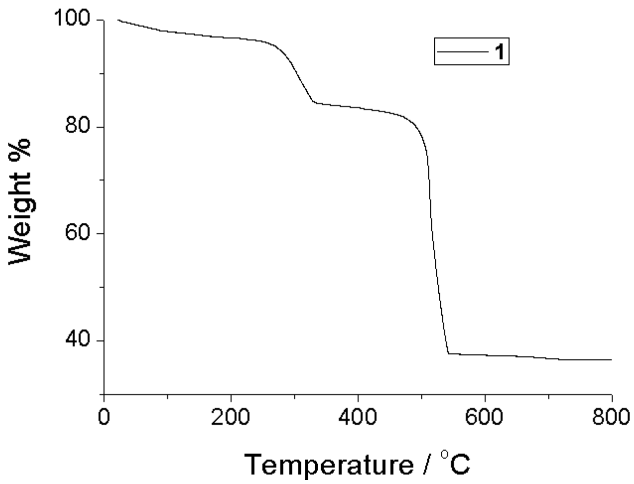

Figure S2. The TGA curve for **1**.

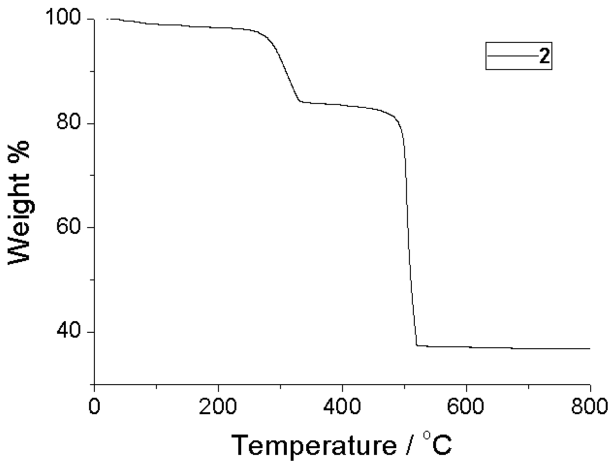

Figure S3. The TGA curve for **2**.
